# Aberrant RhoA activation in macrophages increases senescence-associated secretory phenotypes and ectopic calcification in muscular dystrophic mice

**DOI:** 10.18632/aging.202413

**Published:** 2020-12-23

**Authors:** Xiaodong Mu, Chi-Yi Lin, William S. Hambright, Ying Tang, Sudheer Ravuri, Aiping Lu, Polina Matre, Wanqun Chen, Xueqin Gao, Yan Cui, Ling Zhong, Bing Wang, Johnny Huard

**Affiliations:** 1 Shandong First Medical University and Shandong Academy of Medical Sciences, Jinan, Shandong, China; 2 Steadman Philippon Research Institute, Center for Regenerative Sports Medicine, Vail, CO 81657, USA; 3 Department of Orthopedic Surgery, McGovern Medical School, University of Texas Health Science Center at Houston, Houston, TX 77030, USA; 4 Department of Orthopaedic Surgery, University of Pittsburgh, Pittsburgh, PA 15260, USA; 5 Department of Biochemistry and Molecular Biology, Jinan University, Guangzhou, China

**Keywords:** heterotopic ossification, cellular senescence, muscle dystrophy, muscle stem cell, chronic inflammation

## Abstract

Duchenne Muscular Dystrophy (DMD) patients often suffer from both muscle wasting and osteoporosis. Our previous studies have revealed reduced regeneration potential in skeletal muscle and bone, concomitant with ectopic calcification of soft tissues in double knockout (*dKO*, *dystrophin-/-*; utrophin-/-) mice, a severe murine model for DMD. We found significant involvement of RhoA/ROCK (Rho-Associated Protein Kinase) signaling in mediating ectopic calcification of muscles in *dKO* mice. However, the cellular identity of these RhoA+ cells, and the role that RhoA plays in the chronic inflammation-associated pathologies has not been elucidated. Here, we report that CD68+ macrophages are highly prevalent at the sites of ectopic calcification of *dKO* mice, and that these macrophages highly express RhoA. Macrophages from *dKO* mice feature a shift towards a more pro-inflammatory M1 polarization and an increased expression of various senescence-associated secretory phenotype (SASP) factors that was reduced with the RhoA/ROCK inhibitor Y-27632. Further, systemic inhibition of RhoA activity in *dKO* mice led to reduced number of RhoA+/CD68+ cells, as well as a reduction in fibrosis and ectopic calcification. Together, these data revealed that RhoA signaling may be a key regulator of imbalanced mineralization in the dystrophic musculoskeletal system and consequently a therapeutic target for the treatment of DMD or other related muscle dystrophies.

## INTRODUCTION

Duchenne Muscular Dystrophy (DMD) is caused by mutations of the dystrophin gene and is the most common of the muscular dystrophies [1]. Dystrophin deficiency promotes progressive muscle fiber damage and degeneration, resulting in cardiac or respiratory failure and ultimately premature death. This disease has been well described as a skeletal muscle disease, but DMD also has a significant impact on other musculoskeletal tissues [2, 3]. Osteoporosis, the exhibition of reduced mineral density in bone, is a problem among DMD patients leading to increased incidence of bone fracture [2]. In most DMD animal models, ectopic calcification of soft tissues was also observed [4], highlighting a perturbed balance of mineralization in the disease. This contradictory phenomenon also known as the ‘calcification paradox’ describes the increase in mineralization leading to ectopic calcification with a coincident decrease in mineralization attributed to osteoporosis [5].

Ectopic calcification can form in the soft tissues following trauma, surgery, neurological injury, or genetic abnormalities [6]. Such calcification has also been reported in the skeletal muscles of the telomerase-deficient *mdx* (*mdx*/mTR) mouse model of DMD and Golden retriever muscular dystrophy (GRMD) dogs [7, 8]. Recent publications have demonstrated that there was also extensive ectopic calcification of skeletal and cardiac muscles in the dystrophin/utrophin double knockout [*dKO*, *mdx*:utr(-/-)] model [9, 10]. Thus, ectopic calcification is a phenotype of DMD in several preclinical models. However, the *dKO* model provides a more clinically relevant DMD model versus others given the more severe and accelerated disease progression that better recapitulates pathology seen in humans [11]. Even with the availability of accurate animal models, there is limited examination of the cellular and molecular mechanisms driving ectopic calcification during DMD pathogenesis.

Chronic inflammation, a contributing factor to ectopic calcification [4], is also involved in the dystrophic process, representing a critical pathogenic mechanism in DMD [12, 13]. Pathologies associated with chronic inflammation include tissue degeneration, cell apoptosis, and fibrosis. Glucocorticoids, a well-known anti-inflammatory steroid, are considered the “gold standard” for palliative therapy among those afflicted with DMD [14]. In fact, the administration of various anti-inflammatory medications has been shown to prevent ectopic calcification [15, 16]. However, standard glucocorticoid administration can also have an adverse effect on bone health when treated chronically as shown in the treatment of various types of disease [17–19]. Preclinical DMD models have displayed glucocorticoid-induced osteoporosis and calcification in muscles and other soft tissues [20, 21]. In agreement, we recently showed that although glucocorticoid treatment of *dKO* mice could reduce inflammation and alleviate the muscle stem cell exhaustion/depletion, it also induced ectopic calcification in skeletal muscle of the mice [10]. Therefore, the prevention of glucocorticoid-induced osteoporosis and soft tissue calcification might improve the beneficial effects of this treatment, the most effective clinical therapy for DMD.

Our previous work has demonstrated the critical role of the RhoA/ROCK (Rho-associated kinase) signaling pathway in mediating ectopic calcification in dystrophic skeletal muscles of *dKO* mice [9, 10]. RhoA is a small GTPase involved in the regulation of F-actin stress fiber formation, cell morphology, migration, proliferation, and differentiation of various types of cells [9, 22, 23]. RhoA is also involved in regulating myocardial and pulmonary fibrosis [24], and sustained activation of the RhoA pathway leads to inhibited differentiation of skeletal muscle stem cells [25–27]. Increased cellular calcium influx in various cell types activates RhoA signaling [28, 29]. Importantly, RhoA signaling has been shown to regulate podosome assembly, polarization, function and survival of osteoclast cells [30–32], suggesting a potential mechanism for RhoA in regulating osteoporosis in DMD mice. Recent studies further demonstrated the close association of RhoA signaling with the pro-inflammatory TNF-α/NF-kB signaling [33], and we recently found that RhoA expression is co-activated with pro-inflammatory cytokines TNF-α and IL-6 in both skeletal and cardiac muscle of DMD mice demonstrating that RhoA activation is associated with chronic inflammation in dystrophic muscle [9, 10]. It is also currently unknown if aberrant RhoA activation is present in human DMD patients.

Senescent cells contribute to age-related inflammation via secreting multiple pro-inflammatory factors, which are part of senescence-associated secretory phenotype (SASP) [34, 35]. Senescent macrophages are in fact also found to express senescence-related markers p16(Ink4a) and β-galactosidase (β-gal) and promote inflammation in pathological diseases [36–38]. Our previous work has indicated increased cellular senescence in dystrophic muscles of *dKO* mice [39], however, whether or not macrophages in particular promote senescence, or senescence-associated phenotypes, is currently unknown. To this end, we examined if macrophages from *dKO* mice might develop senescence-associated phenotypes in dystrophic muscle, and whether RhoA/ROCK signaling is involved in the process. We report that skeletal muscle macrophages in dystrophic mice highly express SASP in a RhoA dependent manner suggesting RhoA inhibition may represent a therapy to reduce or restore the unbalanced calcification between soft tissues and bone in *dKO* mice and potentially DMD patients.

## RESULTS

### Increased macrophage and RhoA expressing cells at the site of ectopic calcification and at the bone-muscle interface of *dKO* mice

**Figure 1:**
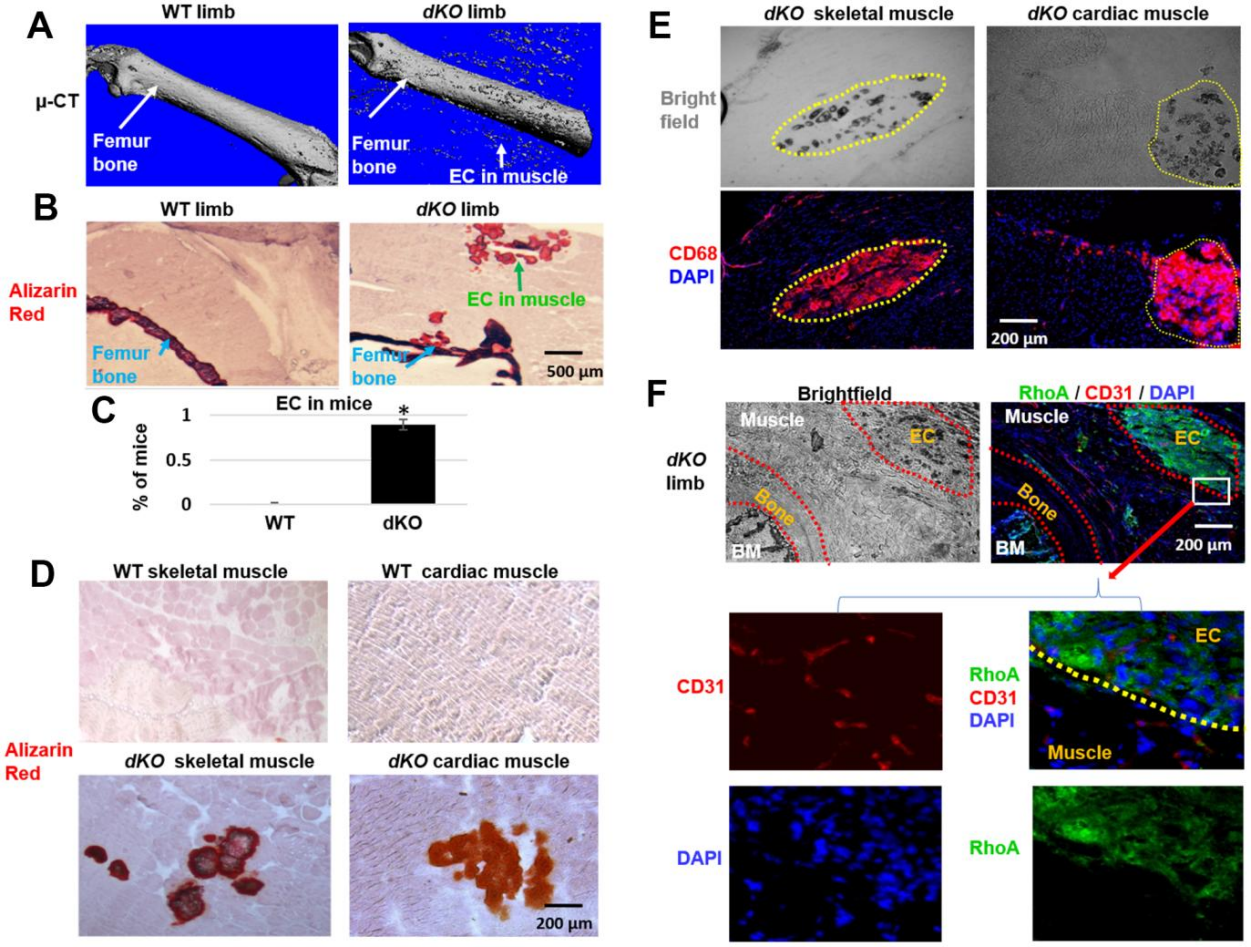
**Increased macrophage accumulation at sites of ectopic calcification in skeletal muscle of *dKO* mice.** (**A**). Micro-CT scanning results indicating increased ectopic calcification and osteoporosis in the hindlimb of *dKO* mice, compared to WT mice. (**B**) Alizarin Red staining of hindlimb tissue sections validate the presence of ectopic calcification in *dKO* mice. (**C**) Percent of mice that exhibit ectopic calcification (EC) in hindlimbs of *dKO* mice and WT mice. (**D**) Alizarin Red staining of skeletal muscle (gastrocnemius) and cardiac muscle (septum) showing ectopic calcification (EC). (**E**) Immunostaining of *dKO* skeletal muscle and heart sections with CD68 antibody and bright field imaging showing the extensive accumulation of CD68+ macrophages at the sites of ectopic calcification (EC). (**F**) Immunostaining of *dKO* hindlimb sections with RhoA and CD31 antibodies and bright field imaging showing the increased accumulation of RhoA+ cells at the sites of ectopic calcification (EC). n=8 for both WT and dKO mice (8-week old).*=*p*<0.05.

*dKO* mice are known to exhibit significant skeletal muscle pathology in addition to loss of bone volume and bone density by 6 weeks of age [11]. To examine characteristics of ectopic calcification in the setting of dystrophic muscle, 8 week old *dKO* mice were sacrificed and hindlimbs were collected for micro-CT and histological analyses. The combination of micro-CT scanning and Alizarin Red staining demonstrated severe osteoporosis and ectopic calcification in the hindlimb muscles adjacent to the femur of *dKO* mice (Figure 1A–1C). Alizarin Red staining also showed the occurrence of ectopic calcification in both skeletal muscle and cardiac muscle of *dKO* mice, but not in WT mice (Figure 1D) (Supplementary Figure 1). These observations indicate a potential correlation between the progression of osteoporosis and muscle tissue calcification in this *dKO* animal model. Immunostaining for CD68+ macrophages in the gastrocnemius muscle and cardiac muscle of the *dKO* mice also demonstrated that macrophages had accumulated extensively at the sites of calcification in both skeletal muscle and cardiac muscle (Figure 1E). The enrichment of CD68+ macrophages at these calcified locations compared to non-calcified locations indicates a potential role of macrophages in regulating ectopic calcification and contributing to the muscle pathology in *dKO* mice.

Our previous work revealed increased RhoA expression in skeletal muscle of *dKO* mice [9, 10]. Here, immunostaining of hindlimb sections of *dKO* mice further demonstrate that RhoA expressing cells also accumulated at ectopic calcification sites in the skeletal muscle (Figure 1F). Similarly, extensive accumulation of RhoA+ cells was also observed in the ectopic calcification sites in cardiac muscle of *dKO* mice (Supplementary Figure 2). The enrichment of RhoA expressing cells at these locations indicates a potential role of RhoA activation in promoting ectopic calcification and affecting the musculoskeletal system of *dKO* mice.

### RhoA activation occurs in CD68+ macrophages that accumulate at the sites of ectopic calcification and at the muscle/bone interface in skeletal muscle of *dKO* mice

**Figure 2:**
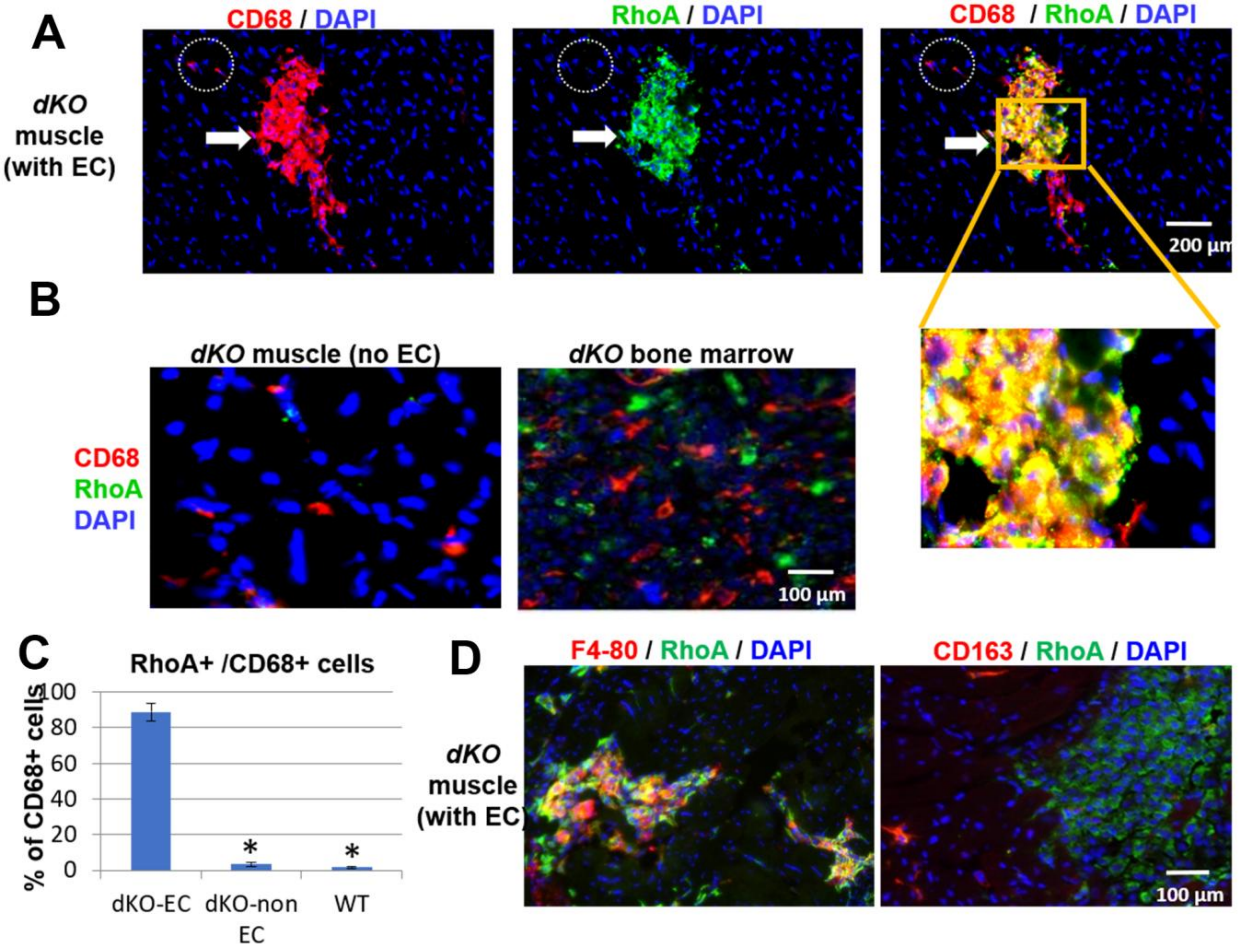
**CD68+ cells at the sites of muscle calcification in *dKO* mice are positive for RhoA expression.** (**A**) Immunostaining of *dKO* skeletal muscle (gastrocnemius) sections with RhoA and CD68 antibodies indicating that many of the accumulated CD68+ cells at sites of ectopic calcification (EC) are also RhoA+. (**B**) Immunostaining of *dKO* skeletal muscle and bone marrow sections with RhoA and CD68 antibodies showing that CD68+ cells distant from ectopic calcification or those found in bone marrow are negative for RhoA expression. (**C**) Immunostaining of *dKO* skeletal muscle with RhoA/F4-80 or RhoA/CD163 antibodies indicating RhoA+ cells are usually positive for F4-80, but negative for CD163 (a marker for M2 macrophages). (**D**) Quantification of RhoA+/CD68+ cells among CD68+ cells at locations of muscle with or without EC formation. * indicates p<0.05.

Since both CD68+ cells and RhoA+ cells accumulated at the same locations (ectopic bone and interface of bone and skeletal muscle) in dystrophic muscles of *dKO* mice, we next examined whether RhoA+ signal colocalized with CD68+ macrophages. Co-immunostaining of RhoA and CD68 showed that many of CD68+ cells accumulated at the sites of ectopic calcification were also highly positive for RhoA expression (Figure 2A), suggesting the cellular identity of RhoA+ cells to be primarily CD68+ macrophages. Interestingly, macrophages found in the locations without ectopic calcification in the muscle or bone marrow were primarily negative for RhoA expression (Figure 2B). Therefore, increased RhoA expression is generally specific to CD68+ cells at the ectopic calcification sites in *dKO* mice (Figure 2C). To further confirm, we observed that RhoA+ cells at the degenerative locations are mostly positive for another macrophage marker, F4-80 (Figure 2D). Interestingly, cells expressing CD163 (a M2 macrophage marker) were found to be distinct from cells with RhoA expression (Figure 2D), indicating that these RhoA+/CD68+ cells were likely activated M1 macrophages. In addition, among all RhoA+ cells in the muscle, we have observed that over 80% of RhoA+ cells were CD68+, while only ~4% of RhoA+ cells were Pax7+ (Supplementary Figure 3). This observation collectively suggest that only those macrophages accumulating at degenerating location with ectopic calcification in muscles are RhoA positive and of the pro-inflammatory M1 phenotype.

### RhoA activation occurs in CD68+ macrophages that accumulate at sites of calcification in cardiac muscle of *dKO* mice

**Figure 3:**
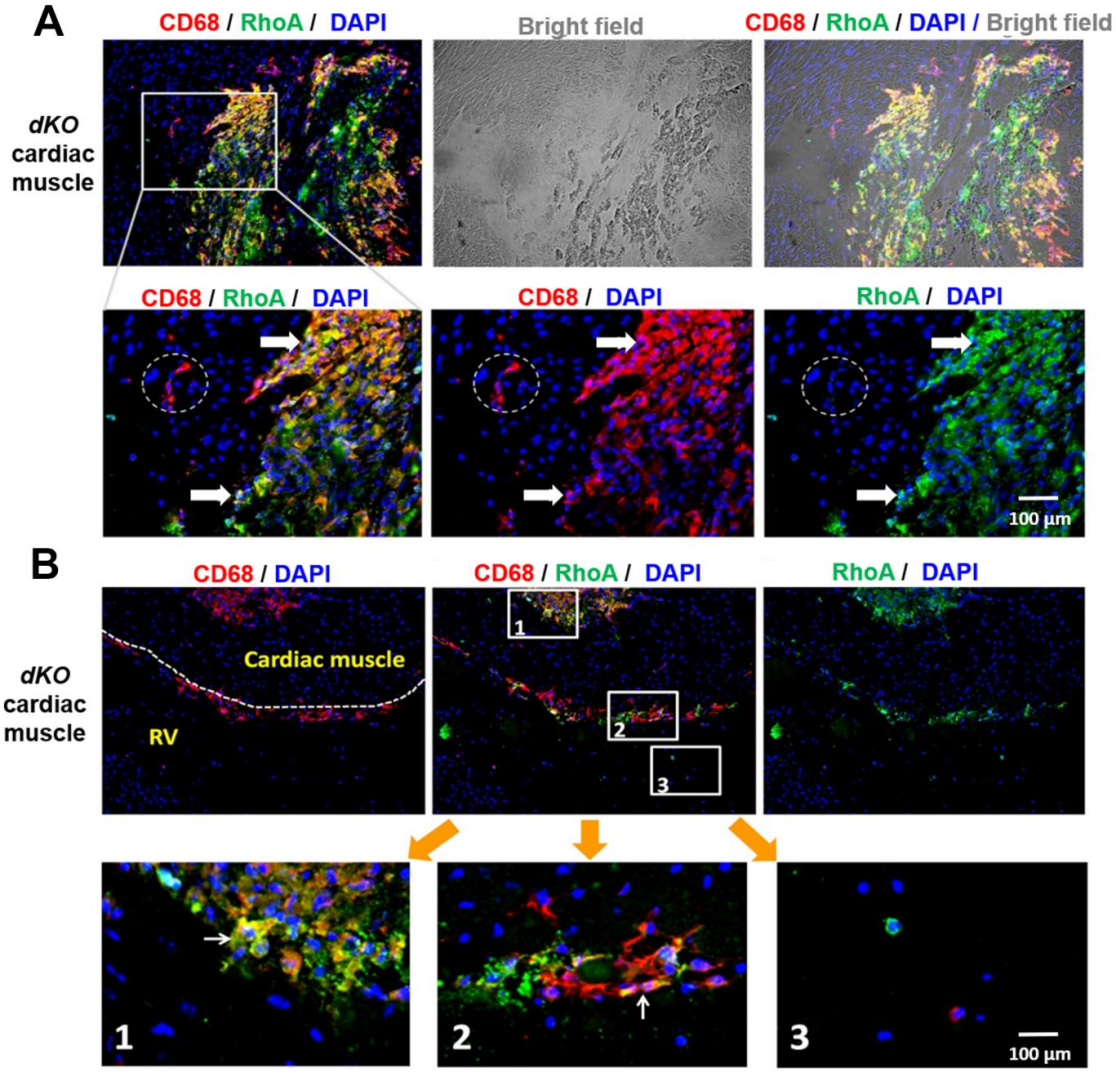
**CD68+ cells at calcification sites in the heart of *dKO* mice co-express RhoA.** (**A**) Immunostaining of *dKO* heart sections (cardiac muscle at septum) with RhoA and CD68 antibodies showing that many of the accumulated CD68+ cells at the sites of ectopic calcification are RhoA+ (white arrows); whereas the CD68+ cells away from the calcification sites are RhoA- (dotted circle). (**B**) Immunostaining of *dKO* heart sections (interface of right ventricle and septum) with RhoA and CD68 antibodies showing accumulation of RhoA+/CD68+ cells at the endothelial barrier (cardiac endothelium, dotted lines) which are infiltrating through the endothelium from circulation towards the pathological area of the cardiac muscle (sub-image 2); whereas the CD68+ cells in the circulating blood are RhoA- (sub-image 3). N=6 for both WT and *dKO* mice (8-week old).

We next evaluated whether CD68 and RhoA expressing cells are also present at the calcification sites in cardiac muscle, muscle tissue not directly adjacent to bone. As seen in skeletal muscle, co-immunostaining of cardiac muscle with antibodies targeting RhoA and CD68 revealed that CD68+ cells at or around calcification sites were RhoA+ (arrows, Figure 3A), while the CD68+ cells distant from the calcification sites were RhoA- (dotted circles, Figure 3A) (Supplementary Figure 4). Similar to skeletal muscle, CD68+/RhoA+ cells accumulated at ectopic calcification sites in the heart (Figure 3A; Figure 3B, sub-image 1). Accumulation of CD68+/RhoA+ cells was also observed at the endothelial barrier (cardiac endothelium, dotted lines, Figure 3B, sub-image 2), which suggests that these CD68-positive cells could be infiltrating through the endothelium from circulation towards the pathological area of the cardiac muscle, which might also be mediated by RhoA activation. In support of this, CD68-positive cells in the circulating blood were found to be RhoA negative (Figure 3B, sub-image 3). Similar to that observed with skeletal muscle, our results suggest that RhoA activity is required for the migration of inflammatory macrophages to the calcification site in cardiac muscle, a phenomenon that has been observed [40], but not yet in DMD.

### Increased expression of SASP factors in macrophages of *dKO* mice is RhoA dependent

**Figure 4:**
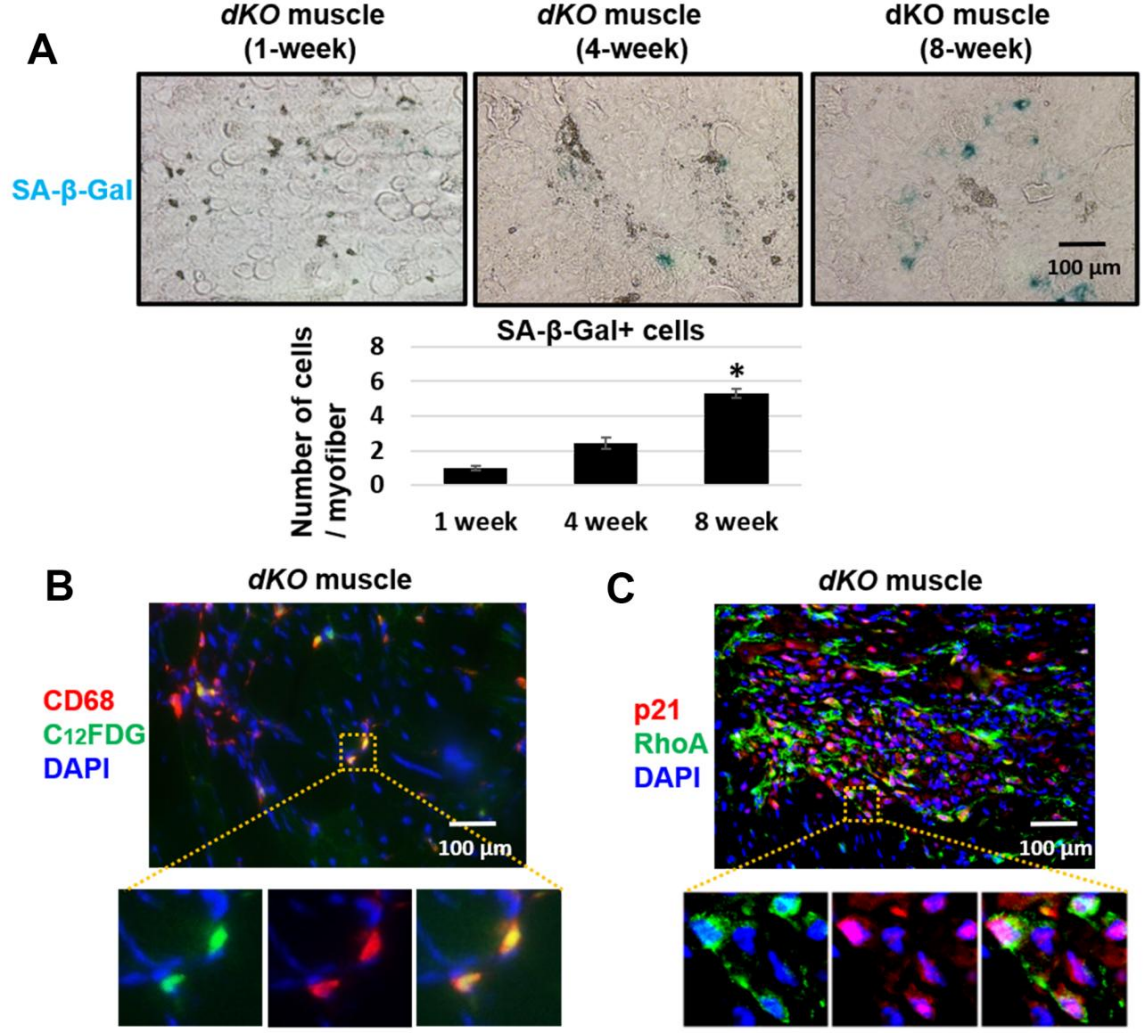
**Macrophages accumulating in dystrophic muscles have increased expression of senescence markers.** (**A**) SA-β-Gal staining of gastrocnemius muscle sections from 1, 4, and 8 week old *dKO* mice with quantification. Significant changes in SA-β-Gal staining was observed by 8 weeks. (**B**) Immunofluorescent staining of gastrocnemius muscle sections from 8-week old *dKO* mice indicating co-localization of the senescence marker C_12_FDG and CD68. ~26% of CD68+ cells are C_12_FDG+. (**C**) Immunofluorescent staining of muscle sections from 8 week old *dKO* mice indicating co-localization of the senescence marker p21 and RhoA. ~78% of RhoA+ cells are p21+. n=6 for 1-week and 4-week old mice, and n=8 for 8-week old mice. * indicates *p*<0.05.

**Figure 5:**
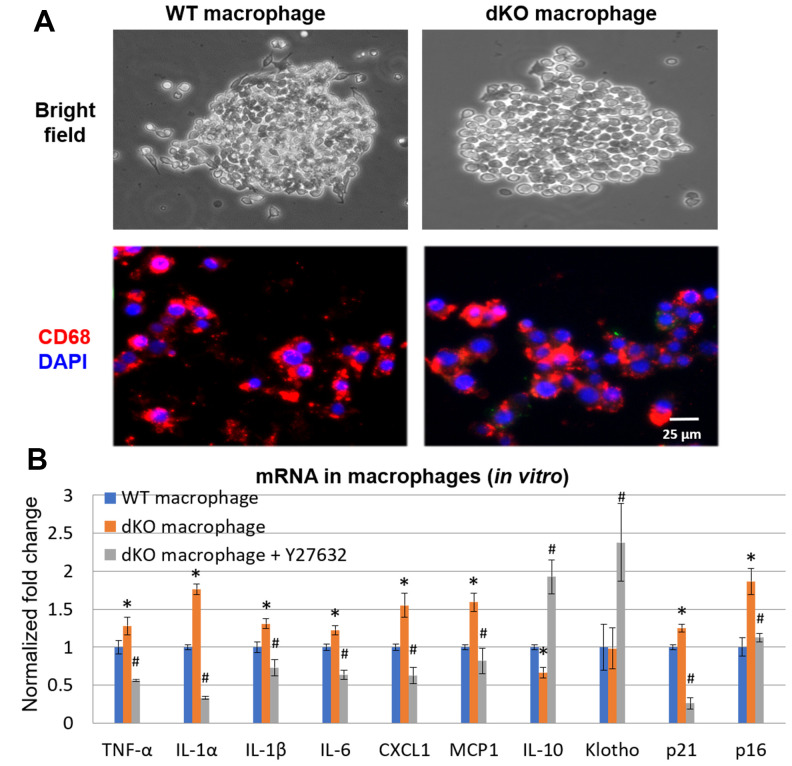
***In vitro* inhibition of RhoA/ROCK in *dKO* macrophages reduced expression of SASP factors.** (**A**) Cultured macrophages isolated from WT and *dKO* mice obtained by FACS with CD68 immunostaining to validate macrophage isolation *in vitro*. (**B**) qPCR results indicating *dKO* macrophages exhibit up-regulated expression of SASP factors that was reduced by treatment with the RhoA/ROCK inhibitor Y-27632 (10 μM, 2 days). * indicates p<0.05 vs. WT, * indicates p<0.05 vs. *dKO*.

Skeletal muscle of *dKO* mice is known to harbor senescent cells [41–45]. A hallmark of senescent cells is the production of a senescence associated secretory phenotype (SASP) [46]. To evaluate whether infiltrating CD68+/RhoA+ macrophages were potentially promoting the histopathology in the skeletal muscle of *dKO* mice through SASP production, we performed immunostaining against senescence-associated-β-galactosidase (SA-β-gal) in the skeletal muscles from 1-, 4-, and 8-week old *dKO* mice. Our results indicate that cellular senescence is increased in the skeletal muscle with advancing age in *dKO* mice (Figure 4A). Co-staining of dystrophic muscle with CD68 antibody and C_12_FDG (a galactosidase substrate and senescence marker) [47] revealed that around 26% of the CD68+ macrophages were positive for C_12_FDG as well (Figure 4B). Co-staining of dystrophic muscle with p21 and RhoA antibodies further revealed that around 78% of the RhoA+ cells were also co-expressing p21 (Figure 4C). Because we have observed that CD68-positive macrophages accumulating at the degenerative sites are mostly RhoA-positive, we posit that RhoA activation in macrophages may lead to increased expression of senescence-related (SASP) factors. To measure SASP production in macrophages more directly, macrophages were isolated from dystrophic muscles of WT and *dKO* mice (8-week old) by Fluorescence activated cell sorting (FACS) of CD68+ cells (Supplementary Figure 5). Macrophages were cultured *in vitro* and immunostaining with CD68 antibody was performed to validate the macrophage phenotype (Figure 5A). RT-PCR analysis revealed that *dKO* macrophages express more SASP factors (i.e., TNFα, IL-1α, IL-1β, IL-6, MCP1, and CXCL1) compared to WT macrophages (Figure 5B). Accordingly, compared to WT macrophages there was about a 1.3-fold increase in the relative mRNA expression of p21, and a 1.9-fold increase for p16 in *dKO* macrophages; whereas the mRNA expression of the anti-inflammatory factor IL-10 was down-regulated (~1.5-fold). Since SASP factors are known to be dominant in M1 macrophages [48], it is likely that these RhoA+ macrophages are primarily M1 polarized pro-inflammatory macrophages.

Finally, to determine if RhoA mediated SASP production in macrophages from *dKO* mice, *dKO* macrophages were isolated and treated with the RhoA/ROCK inhibitor Y-27632 (10 μM) for 48 hr. RT-PCR analysis showed that RhoA/ROCK inhibition in *dKO* macrophages repressed the expression of SASP factors TNFα, IL-1α, IL-1β, IL-6, MCP1, and CXCL1 (Figure 5B). Further, the expression of p21 was also reduced, and the anti-inflammatory factors IL-10 and Klotho were also increased following Y-27632 treatment. Thus, RhoA seems to mediate pro-inflammatory SASP production in macrophages associated with muscle pathology in *dKO* mice.

### *In vivo* inhibition of RhoA/ROCK in *dKO* mice reduces calcification by repressing the accumulation of RhoA+/CD68+ M1 macrophages

**Figure 6:**
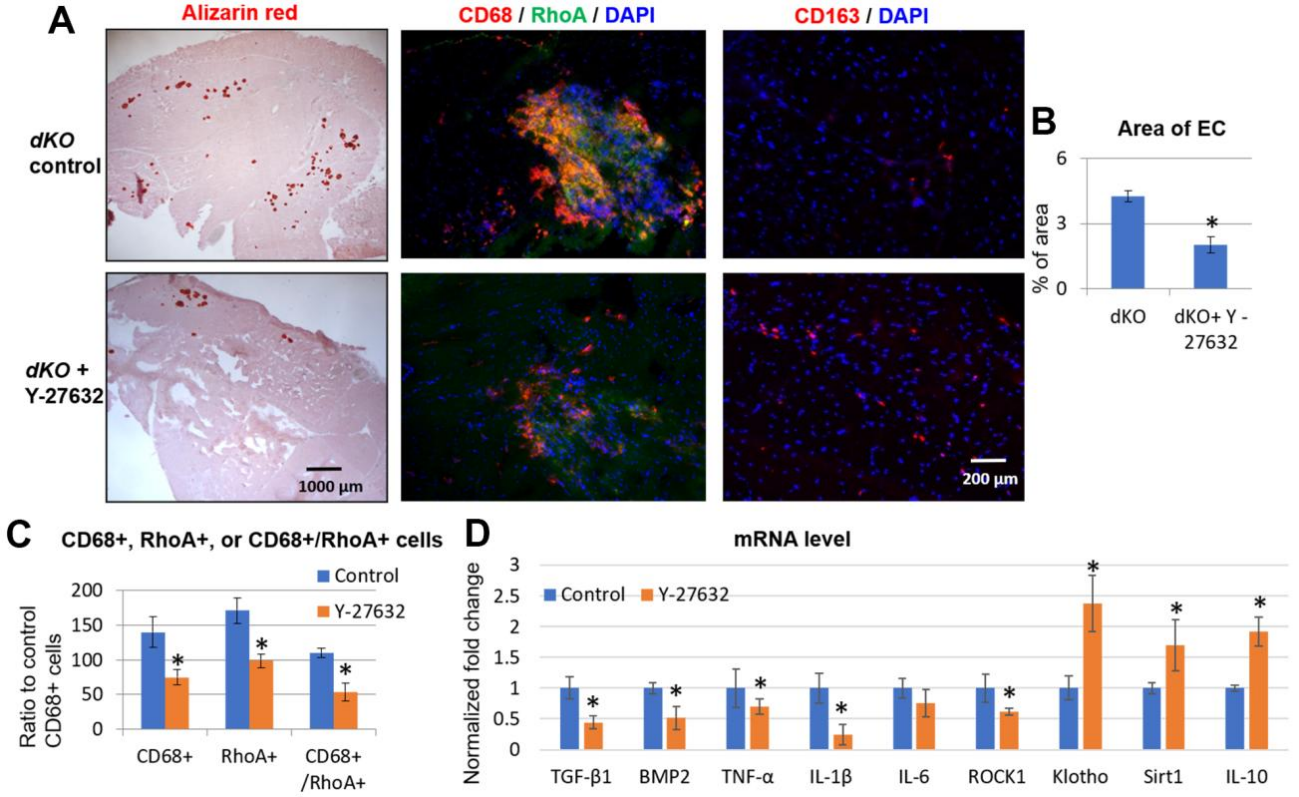
**Systemic inhibition of RhoA/ROCK in *dKO* mice reduced calcification in skeletal muscle by repressing the accumulation of RhoA+/CD68+ cells.** (**A**) Alizarin Red staining indicating reduction in calcification in dystrophic muscles of *dKO* mice treated with the RhoA/ROCK inhibitor Y-27632 for 3 times a week, from 3 weeks to 8 weeks of age. Immunostaining of skeletal muscle tissues showed that the accumulation of CD68+/RhoA+ cells in *dKO* muscle was also reduced by RhoA/ROCK inhibition, while the number of CD163+ cells (M2 macrophages) was increased. (**B**) quantification of the area of ectopic calcification (EC) in skeletal muscle of *dKO* mice with or without Y-27632 treatment. (**C**) Quantification of the number of CD68+, RhoA+, and CD68+/RhoA+ cells with and without Y-27632 treatment (number of cells per area of 100 myofibers). (**D**) qPCR results of mRNA isolated from dystrophic muscles of *dKO* mice showing that Y-27632 treatment significantly down-regulated the expression of pro-inflammatory/fibrosis genes (TGF-β1, BMP2, TNF-α, and IL-1β), and up-regulated the expression of anti-inflammatory/fibrosis genes (Klotho, Sirt1, and IL-10). n=8 for both dKO mice with or without Y-27632 treatment, * indicates *p*<0.05.

To determine if RhoA inhibition could rescue the dystrophic phenotypes *in vivo*, we next inhibited RhoA signaling systemically in *dKO* mice via intraperitoneal (IP) injection of Y-27632, 3 times a week, from 3 weeks to 8 weeks of age, as previously described [9]. Our results demonstrated that calcification in dystrophic muscles was reduced with RhoA inhibition (Figure 6A, 6B). It is notable that, not only was the number of RhoA+ cells reduced in dystrophic muscle when RhoA was inhibited, but the numbers of CD68+ cells and CD68+/RhoA+ cells were also reduced (Figure 6A, 6C), which correlates with a reduction in ectopic calcification in dystrophic muscles. In addition, the number of CD163+ cells (M2 macrophages) were also increased after Y-27632 treatment (Figure 6A). The increased ratio of CD163+ cells to CD68+ cells (all macrophages) after Y27632 treatment suggests that RhoA might mediate the M1 to M2 phase transition of macrophages in *dKO* muscles. Moreover, mRNA isolated from dystrophic muscles of *dKO* mice showed that Y-27632 treatment down-regulated the expression of pro-inflammatory/fibrosis genes (i.e., those expressing TGF-β1, BMP2, TNF-α, IL-1β, and IL-6) in *dKO* muscle, and up-regulated the expression of anti-inflammatory/fibrosis genes (i.e., those expressing Klotho, Sirt1, and IL-10) (Figure 6D). Similarly, *in vivo* inhibition of RhoA/ROCK signaling in *dKO* mice also improved dystrophic phenotypes in cardiac muscle (Supplementary Figure 6). These data were confirmed histologically as well after Y-27632 treatment of *dKO* mice. Alizarin Red staining revealed a reduction in calcification and trichrome staining showed a reduction in fibrosis in cardiac tissue (Supplementary Figure 6). Immunostaining also indicated a reduced number of CD68+/RhoA+ cells, and immunostaining with CD163 and RhoA antibodies showed increased number of CD163+/RhoA- cells (Supplementary Figure 6). Taken together, these results suggest a significant role for RhoA in promoting macrophage infiltration into calcification sites and pro-inflammatory activity in *dKO* skeletal and cardiac muscle and consequently the inhibition of RhoA may represent a therapeutic target to improve the muscle pathology observed in DMD patients.

## DISCUSSION

The occurrence of calcification in blood vessels and other soft tissues coincident with decreased bone mineral density, known as the “calcification paradox”, can be found in various chronic diseases, including DMD patients who suffer from development of both muscle wasting and osteoporosis. Ectopic calcification (EC) in soft tissues is also often associated with chronic inflammatory diseases [4, 49], and ectopic calcification has been described in the skeletal muscles of almost all animal models for human DMD [8, 50]. Our previous work has demonstrated that there exists extensive ectopic calcification in the skeletal muscle of *dKO* mice, and that the activation of RhoA/ROCK signaling was critical in the process [9, 10]. Here, we furthered these data by demonstrating that CD68+ macrophages were highly prevalent at the sites of degeneration and ectopic calcification that were predominantly positive for RhoA expression. Further, we found that infiltrating CD68+/RhoA+ macrophages in *dKO* mice featured increased expression of cellular senescence-associated cell markers (p21, p16, and C_12_FDG) and SASP factors. We also found that the systemic inhibition of RhoA in *dKO* mice was effective in reducing the number of RhoA+/CD68+ cells in dystrophic skeletal muscle and heart, leading to improved muscle histopathology in *dKO* mice. Accordingly, RhoA inhibition reduced the expression of SASP factors in macrophages and promoted the transition of M1 (pro-inflammatory) to M2 (anti-inflammatory) macrophages.

Our findings implicate a novel role of RhoA in regulating the activation and function of macrophages that promote the histopathology and muscular dystrophy in *dKO* mice. Importantly, RhoA activation in macrophages seems to be closely coupled with increased ectopic calcification. The role of RhoA in directly regulating macrophage migration and function has been described [51, 52]. Also, RhoA can be involved in the process of differentiation and polarization of macrophages [53, 54]. However, it is still unclear whether macrophage changes are directly responsible for mineralization in muscle, and whether the M1 vs. M2 status of macrophages is a cause or a consequence of RhoA activation and mineralization changes. We thus postulate that RhoA activation potentially regulates macrophage recruitment, trafficking, and/or polarization during the process of ectopic calcification in dystrophic muscle. Moreover, since SASP factors are known to be dominant in M1 macrophages [48], it is likely that these RhoA+ macrophages are primarily M1 polarized pro-inflammatory macrophages.

In one interesting report using bovine VSMCs, it was found that the RhoA/ROCK signaling pathway is an important negative regulator of vascular calcification [55], while the inhibition of RhoA/ROCK improved the calcification capacity of the VSMCs. Thus, the potential role of RhoA activation in different cell types may have a varied impact on soft tissue calcification. Unlike VSMCs, macrophages cannot directly differentiate into osteogenic cells thus it stands to reason that the potential function of macrophages in regulating ectopic calcification in dystrophic muscle is likely mediated by their secreted factors and their impact on other cell types at the degenerative location. Osteogenic progenitor cells have been shown to differentiate into bone tissue during ectopic bone formation in soft tissue [56] and macrophages are known to fuse and form CD68+ osteoclasts under certain pro-osteoclastic microenvironments [57]. Further, RhoA activation is critical for podosome assembly, polarization, function and survival of osteoclast cells [30–32]. Here, we have observed RhoA activation in some of CD68+ cells accumulating at the pathological sites, suggesting a potential role for RhoA in activating osteoclasts, mediating osteoporosis of the bone tissue and promoting inflammation in *dKO* mice. Along these lines, systemic inhibition of RhoA signaling with the RhoA/ROCK inhibitor Y-27632 in *dKO* mice reduced ectopic calcification which appeared to act, at least in part, by regulating the function of macrophages. Thus, repression of RhoA-mediated macrophage accumulation, and associated macrophage derived SASP factors, at pathological sites (ectopic bone formation) may therefore reduce the recruitment of osteogenic progenitor cells from the bone to dystrophic muscle. In addition, because RhoA inhibition is known to improve the myogenic potential of skeletal muscle progenitor cells [25–27], it might also be that reduced RhoA activation in muscle progenitor cells may in fact promote their myogenic potential and muscle regeneration.

Taken together, our results reveal that accumulation of RhoA-expressing cells, mainly pro-inflammatory macrophages, are involved in the calcification paradox that occurs during disease progression in *dKO* mice, and that RhoA inhibition can restore the imbalanced mineralization (i.e. osteoporosis with co-incident ectopic bone formation in dystrophic muscle tissue) by limiting the accumulation of pro-inflammatory macrophages. These data thus suggest a mechanism whereby RhoA/ROCK activation might be a key regulator of imbalanced mineralization in the musculoskeletal system of DMD or related muscle dystrophies. Inhibition of RhoA could therefore represent a novel therapeutic approach to reduce musculoskeletal pathologies induced by chronic inflammation during the progression of disease in muscular dystrophy.

## MATERIALS AND METHODS

### Animals

WT (C57BL/10J, male) mice were obtained from the Jackson Laboratory (Bar Harbor, ME). *mdx*:utr(-/-) mice (dystrophin/utrophin double knock out, *dKO*, or *dystrophin*^-/-^:utrophin^-/-^; male mice) were derived from our in-house colony. Mice were housed in groups of 4 on a 12:12-hour light-dark cycle at 20-23° C. At least 8 mice were used in each experimental sample group. All procedures were approved by the Institutional Animal Care and Use Committee (IACUC) at the University of Pittsburgh (IACUC-1109718) and the University of Texas Health Science Center at Houston (AWC-18-0068).

### Isolation of macrophages from skeletal muscles

Gastrocnemius (GM) muscle tissues were harvested from WT and *dKO* mice (8-week old), and digested by serial 1-hr incubations at 37° C in 0.2% type XI collagenase (Sigma, Burlington, MA, USA), dispase (grade II, 240 units; Sigma), and 0.1% trypsin (ThermoFisher, Waltham, MA, USA), as previously described [58]. Primary muscle cells were harvested after digestion, and macrophages were then isolated from these primary muscle cells by Fluorescence activated cell sorting (FACS) of CD68+ cells with a cell sorter (BD FACSAria, San Jose, CA, USA). Macrophages were cultured and immunostaining with CD68 antibody was performed to validate the identity of the isolated cells to be macrophages; the primary muscle cells were also immunostained with CD68 antibody to serve as negative control.

### RhoA inhibition with Y-27632 *in vitro* and *in vivo*

*In vitro*: Cultured primary mouse macrophages (1000 cells per well in a collagen-coated 12-well plate) were treated with the RhoA/ROCK inhibitor Y-27632 (EMD Millipore, Billerica, MA, USA) (10 μM) in proliferation medium (PM, 10% FBS in DMEM) for 2 days prior to expression analysis. *In vivo*: Systemic inhibition of RhoA signaling was accomplished by IP injection of Y-27632 (5 mM in phosphate buffered saline [PBS], 10 mg/kg per mouse) into *dKO* mice, starting at 3 weeks of age for 3 times a week for 5 weeks. Controls were PBS injections alone. We did not see inflammatory reactions caused by IP injections in both control and treatment groups. Skeletal muscle (gastrocnemius) and the whole hearts of the mice (n=8) were collected and frozen for further analysis. Gastrocnemius muscles were cryo-sectioned at around 40% - 60% from the top (tendon side), and the hearts were cryo-sectioned at around 20%-40% from the bottom of heart, to obtain slides for immunostaining and histology assays.

### mRNA analysis with reverse transcriptase-PCR

Total RNA was obtained from cells or skeletal muscle of WT and *dKO* mice using the RNeasy Mini Kit (Qiagen, Inc., Valencia, CA, USA) according to the manufacturer’s instructions. Reverse transcription was performed using the iScript cDNA Synthesis Kit (Bio-Rad, Hercules, CA, USA). Primer sequences are shown in Table 1. PCR reactions were performed using an iCycler Thermal Cycler (Bio-Rad). The cycling parameters used for all primers were as follows: 95° C for 10 minutes; PCR, 40 cycles of 30 seconds at 95° C for denaturation, 1 minute at 54° C for annealing, and 30 seconds at 72° C for extension. Products were separated by size and were visualized on 1.5% agarose gels stained with ethidium bromide. All data were normalized to the expression of GAPDH using the ΔΔCt method.

**Table 1:** Primer sequences.

**Gene**	**Primer sequence**
GAPDH	Forward: TCCATGACAACTTTGGCATTG
	Reverse: TCACGCCACAGCTTTCCA
TGF-beta1	Forward: CTCCCGTGGCTTCTAGTGC
	Reverse: GCCTTAGTTTGGACAGGATCTG
TNF-α	Forward: CCTGTAGCCCACGTCGTAG
	Reverse: GGGAGTAGACAAGGTACAACCC
CXCL1	Forward: CTGGGATTCACCTCAAGAACATC
	Reverse: CAGGGTCAAGGCAAGCCTC
IL-1alpha	Forward: TCTCAGATTCACAACTGTTCGTG
	Reverse: AGAAAATGAGGTCGGTCTCACTA
IL-1beta	Forward: GCAACTGTTCCTGAACTCAACT
	Reverse: ATCTTTTGGGGTCCGTCAACT
IL-6	Forward: CTGCAAGAGACTTCCATCCAG
	Reverse: AGTGGTATAGACAGGTCTGTTGG
IL-10	Forward: ATTTGAATTCCCTGGGTGAGAAG
	Reverse: CACAGGGGAGAAATCGATGACA
Klotho	Forward: ACTACGTTCAAGTGGACACTACT
	Reverse: GATGGCAGAGAAATCAACACAGT
MCP1	Forward: TAAAAACCTGGATCGGAACCAAA
	Reverse: GCATTAGCTTCAGATTTACGGGT
BMP-2	Forward:TCTTCCGGGAACAGATACAGG
	Reverse: TGGTGTCCAATAGTCTGGTCA
PDGFR-beta	Forward: CAAGAAGCGGCCATGAATCAG
	Reverse: CGGCCCTAGTGAGTTGTTGT
p21	Forward: AGTGTGCCGTTGTCTCTTCG
	Reverse: ACACCAGAGTGCAAGACAGC
p16	Forward: CGCAGGTTCTTGGTCACTGT
	Reverse: TGTTCACGAAAGCCAGAGCG
ROCK1	Forward: GACTGGGGACAGTTTTGAGAC
	Reverse: ATCCAAATCATAAACCAGGGCA
Sirt1	Forward: GCTGACGACTTCGACGACG
	Reverse: TCGGTCAACAGGAGGTTGTCT

### Histology analysis and immunostaining of tissues

Hind limbs (containing femur and the surrounding skeletal muscles), skeletal muscle, and hearts were harvested from 8-week-old WT and *dKO* mice for histological analyses. Ectopic calcification in tissues was assessed by Alizarin Red staining. Immunofluorescent staining was performed with frozen tissue sections fixed with 4% paraformaldehyde. Primary antibodies specific for RhoA (Santa Cruz, Santa Cruz, CA, USA), CD68 (Abcam, Cambridge, MA, USA), F4-80 (Abcam), CD163 (Santa Cruz), pSmad-5 (Santa Cruz), PDGFR-α (Abcam), CD31 (Abcam), and VWF (Abcam) were applied at 1:100 ~ 1:200. Secondary antibodies conjugated with Alexa-488 pr Alexa-594 (ThermoFisher) was applied at 1:400. Negative control for immunofluorescent staining was performed with applying the secondary antibodies only to the tissue sections. All slides were analyzed via fluorescent microscopy (Nikon Instruments Inc., Shinagawa, Japan) and photographed at 4-40X magnification.

### Quantification of results and statistical analysis

Captured images were analyzed using commercially available software (Northern Eclipse, version 6.0; Empix Imaging, Inc., Mississauga, ON, Canada) and ImageJ software (version 1.32j; National Institutes of Health, Bethesda, MD, USA). Data from at least three samples from each subject were pooled for statistical analysis. Results are given as the mean ± standard error (SE). Statistical significance was determined using the Student’s *t*-test for all pairwise comparisons. *P*<0.05 was considered statistically significant.

## Supplementary Material

Supplementary Figures
