# Supplementary material for: Aberrant RhoA activation in macrophages increases senescence-associated secretory phenotypes and ectopic calcification in muscular dystrophic mice
Source: Aging (Albany NY). 2020 Dec 23;12(24):24853–71. doi: 10.18632/aging.202413 (PMC7803538; doi:10.18632/aging.202413)
Supplement: Supplementary Figures [file aging-12-202413-s001.pdf]

SUPPLEMENTARY FIGURES

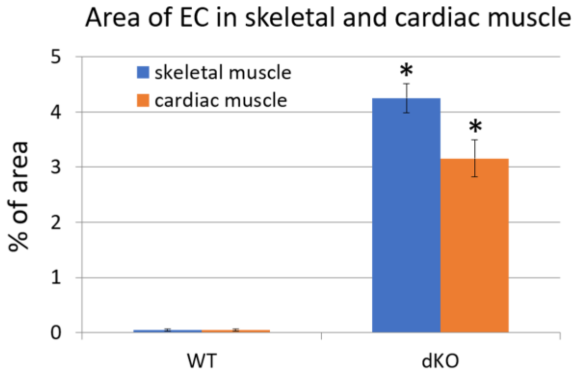

Supplementary Figure 1. Ectopic calcification in skeletal muscle and cardiac muscle of WT and *dKO* mice. Quantification of the area of ectopic calcification in skeletal muscle and cardiac muscle of WT and *dKO* mice. n=6 for both WT and *dKO* mice. "*" indicates $p<0.05$.

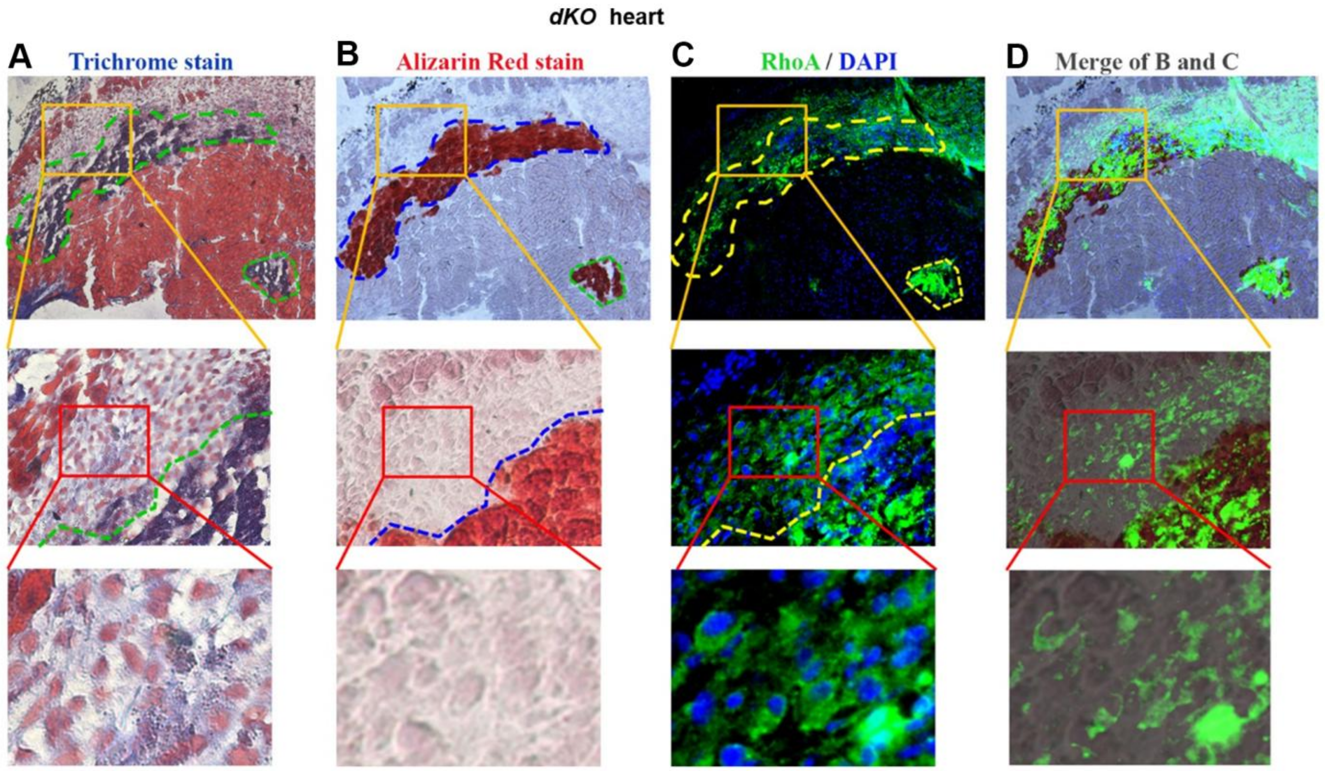

Supplementary Figure 2. Increased accumulation of RhoA expressing cells in ectopic calcification sites in the hearts of *dKO* mice. Trichrome staining (**A**), HE staining (**B**), and immunostaining of *dKO* heart section with RhoA antibody (**C**) showed the increased accumulation of RhoA+ cells at the sites of ectopic calcification. The merged images of B and C is shown in (**D**).

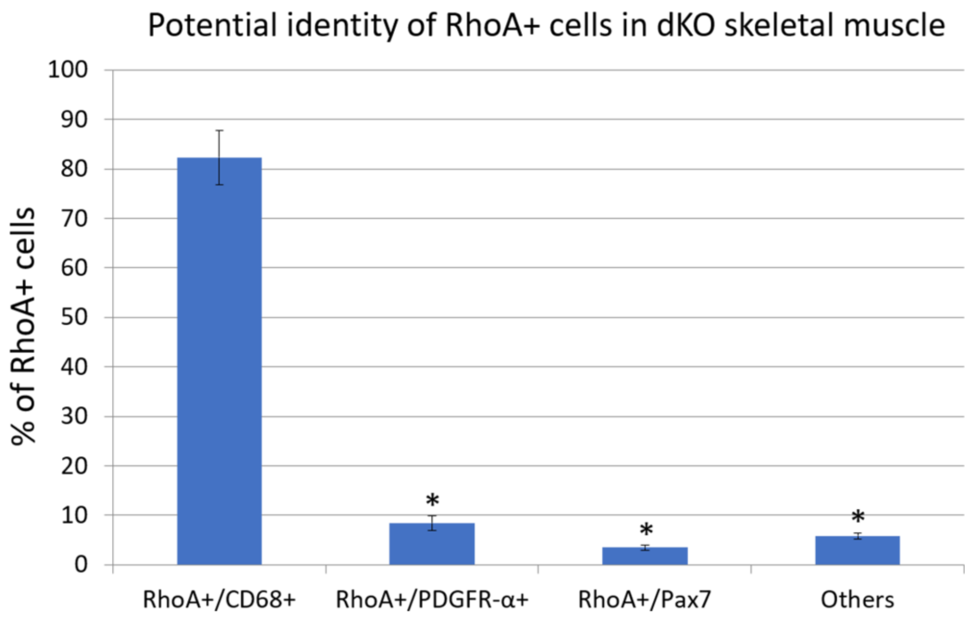

Supplementary Figure 3. Identity of RhoA-expressing (RhoA+) cells in *dKO* skeletal muscle. Quantification of the number of RhoA+ cells positive with CD68 (marker for macrophages), PDGFR-α (marker for mesenchymal stromal cells/MSCs), and Pax7 (marker for muscle progenitor cells). n=6 *dKO* mice (8-week old). "*" indicates $p<0.05$.

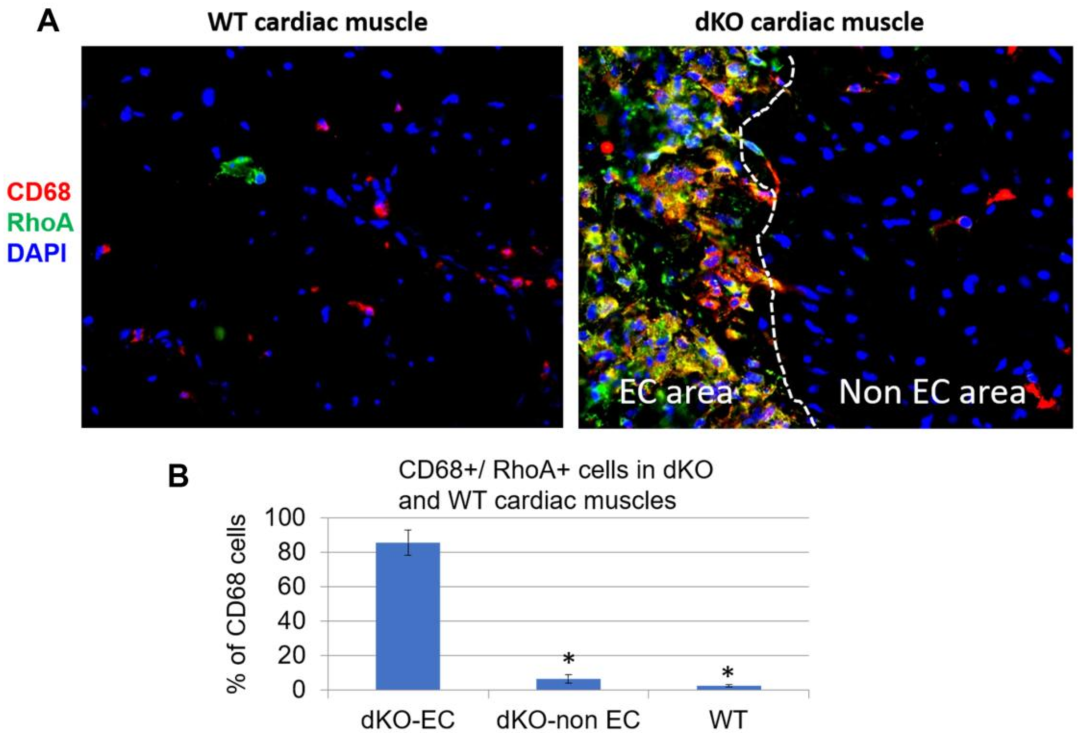

Supplementary Figure 4. CD68+ cells at ectopic calcification sites are positive of RhoA expression. (**A**) Immunostaining of CD68 and RhoA in the cardiac muscles of WT and dKO mice, showing whether CD68+ cells are positive with RhoA expression or not. (**B**) Quantification of the number of CD68+ cells positive of RhoA in dKO muscle with EC, dKO muscle without EC, and WT muscle.. n=6 for both WT and dKO mice. "*" indicates $p<0.05$.

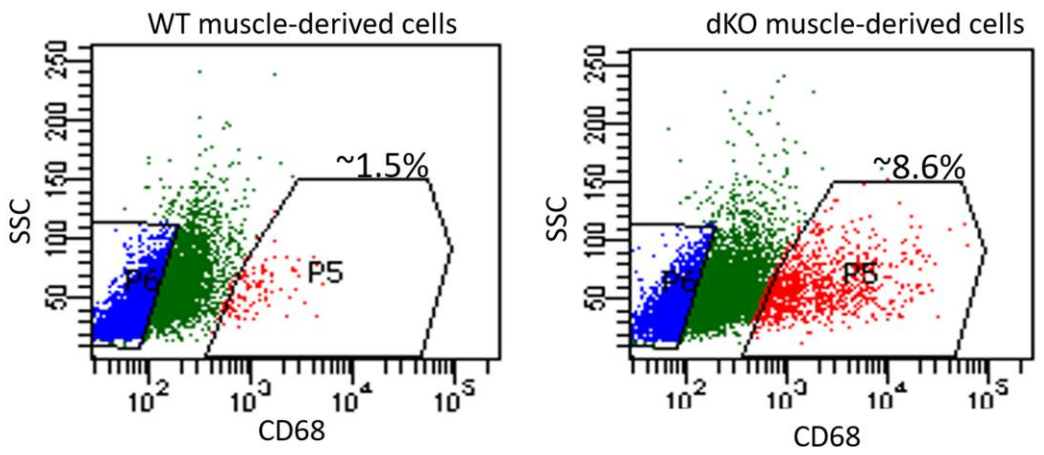

Supplementary Figure 5. Sorting of CD68+ cells from muscle-derived cells of WT and dKO mice. ***Fluorescence*** activated ***cell sorting*** (FACS) of CD68+ cells shows much higher number and ratio of CD68+ cells among all the muscle-derived cells from gastrocnemius muscle of dKO mice, compared to that of WT mice.

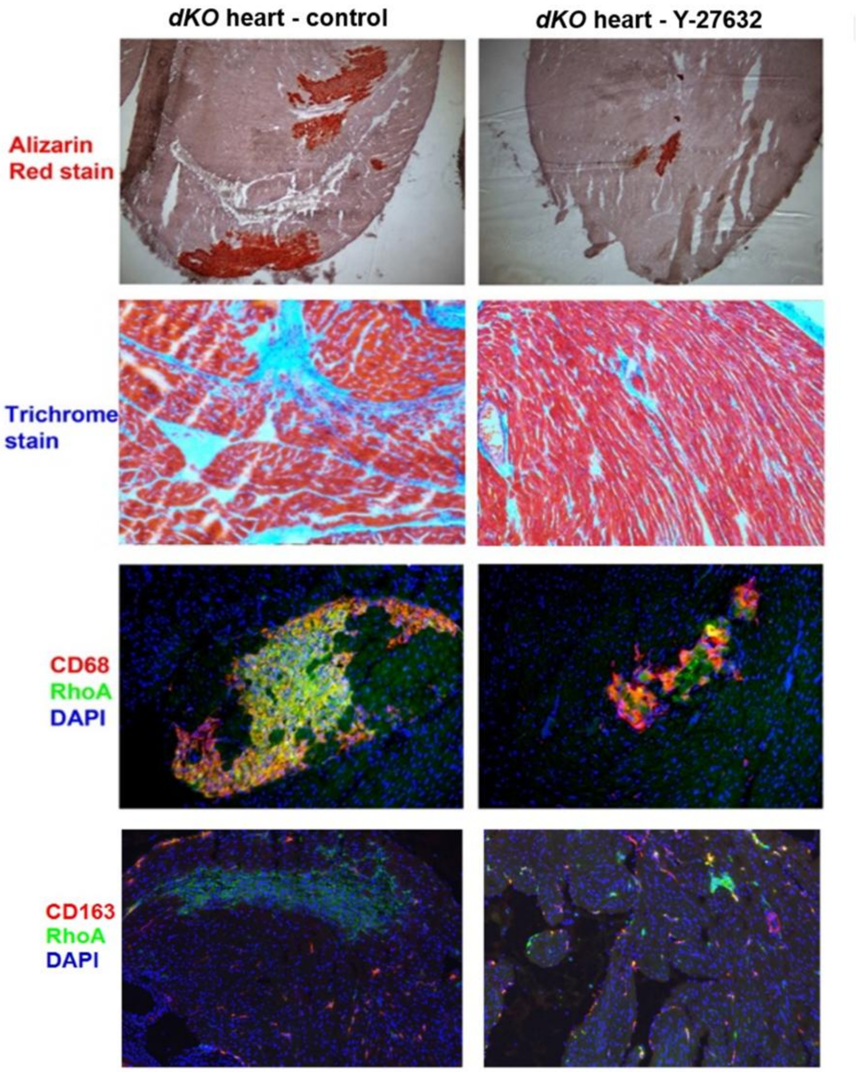

Supplementary Figure 6. *In vivo* inhibition of RhoA/ROCK signaling in dKO mice improved dystrophic phenotypes in hearts. Alizarin Red staining showed reduced calcification in heart tissue with Y-27632 treatment. Trichrome staining showed reduced fibrosis formation in heart tissue with Y-27632 treatment. Immunostaining with CD68 and RhoA antibodies showed reduced number of CD68+/RhoA+ cells after Y-27632 treatment. Immunostaining of cardiac muscle with RhoA- and CD163-specific antibodies revealed that the ratio of CD163+ cells (M2 macrophage) to RhoA+ cells (mainly M1 macrophage) increased after Y27632 treatment, suggesting that the M1 to M2 phase transition of macrophages could have occurred in the Y-27632-treated dystrophic muscles.
